# Bacteriophage-Resistant Mutants in *Yersinia pestis*: Identification of Phage Receptors and Attenuation for Mice

**DOI:** 10.1371/journal.pone.0025486

**Published:** 2011-09-28

**Authors:** Andrey A. Filippov, Kirill V. Sergueev, Yunxiu He, Xiao-Zhe Huang, Bryan T. Gnade, Allen J. Mueller, Carmen M. Fernandez-Prada, Mikeljon P. Nikolich

**Affiliations:** Division of Bacterial and Rickettsial Diseases, Department of Emerging Bacterial Infections, Walter Reed Army Institute of Research, Silver Spring, Maryland, United States of America; Tulane University, United States of America

## Abstract

**Background:**

Bacteriophages specific for *Yersinia pestis* are routinely used for plague diagnostics and could be an alternative to antibiotics in case of drug-resistant plague. A major concern of bacteriophage therapy is the emergence of phage-resistant mutants. The use of phage cocktails can overcome this problem but only if the phages exploit different receptors. Some phage-resistant mutants lose virulence and therefore should not complicate bacteriophage therapy.

**Methodology/Principal Findings:**

The purpose of this work was to identify *Y. pestis* phage receptors using site-directed mutagenesis and *trans*-complementation and to determine potential attenuation of phage-resistant mutants for mice. Six receptors for eight phages were found in different parts of the lipopolysaccharide (LPS) inner and outer core. The receptor for R phage was localized beyond the LPS core. Most spontaneous and defined phage-resistant mutants of *Y. pestis* were attenuated, showing increase in LD_50_ and time to death. The loss of different LPS core biosynthesis enzymes resulted in the reduction of *Y. pestis* virulence and there was a correlation between the degree of core truncation and the impact on virulence. The *yrbH* and *waaA* mutants completely lost their virulence.

**Conclusions/Significance:**

We identified *Y. pestis* receptors for eight bacteriophages. Nine phages together use at least seven different *Y. pestis* receptors that makes some of them promising for formulation of plague therapeutic cocktails. Most phage-resistant *Y. pestis* mutants become attenuated and thus should not pose a serious problem for bacteriophage therapy of plague. LPS is a critical virulence factor of *Y. pestis*.

## Introduction

*Yersinia pestis* is the causative agent of plague circulating in natural foci among multiple species of wild animals. Humans usually become infected via fleabites. Plague has killed many millions of people during three pandemics and is now recognized as a re-emerging disease. *Y. pestis* is considered a biothreat agent belonging to the most dangerous group of pathogens, CDC Category A [1]–[3]. Multiple drug-resistant strains of *Y. pestis* have been isolated from patients with bubonic plague. One of them carried genes of high-level resistance to 12 antibiotics, including virtually all of the drugs commonly used for plague prophylaxis and treatment [4]. The emergence of multidrug-resistant strains requires the immediate development of alternative methods of plague therapy including the possible use of bacteriophages [5]. There are more and more publications on successful bacteriophage treatment of various experimental and veterinary infections and some promising clinical trials in humans (for reviews, see [6]–[8]).

Many bacteriophages (phages) are capable of lysing *Y. pestis* [9]–[21], and some of them have a high potential to be used for antibacterial treatment of plague. Several phages have been shown to be highly lytic and specific for *Y. pestis* and are routinely used for diagnosis of plague [22], [23]. They include the Pokrovskaya [9], [14], [23], [24], ϕA1122 [10], [18], [22], [25], L-413C [14], [19], [23], [24], and Yep-phi [21] phages. The ϕA1122 [26], [27] and L-413C [27] phages have been recently used for the development of improved *Y. pestis* detection techniques using fluorescently labeled ϕA1122 [26] or amplification of phage DNA monitored by qPCR [27]. The genomes of ϕA1122 [18], L-413C [19], and Yep-phi [21] have been sequenced and no genes potentially detrimental for mammals were found in them.

A major concern regarding the use of phages in the treatment of infectious diseases still remains the emergence of phage-resistant mutants [28]–[30]. This resistance can arise due to the alteration or loss of the bacterial cell surface receptor, blocking the receptor by the bacterial extracellular matrix, inhibition of phage DNA penetration, production of modified restriction endonucleases degrading phage DNA, or inhibition of phage intracellular development [31]. Mutations affecting phage receptors represent the most frequent cause of phage resistance [31]–[33]. Phage receptors are diverse bacterial surface-exposed molecules including many outer membrane proteins, sugar residues in the O antigen or lipopolysaccharide (LPS) core, teichoic acids, polysaccharides of the capsule or slime layer, or components of flagella and pili [32], [33]. In pathogenic bacteria, many phage receptors are essential virulence factors, some of which were identified through the selection of phage-resistant mutations. The receptors include capsular polysaccharides [34]–[36], adhesion and invasion factors [37]–[39], a protein involved in intracellular growth [40], and, very commonly, different components of LPS [41]–[47]. If a phage receptor lies in a surface structure important for virulence, the phage-resistant mutants resulting from a loss or alteration of the receptor will be avirulent or attenuated. Such mutant bacterial clones will then be eliminated from the host by the immune system and therefore should not present a problem if they arise during bacteriophage treatment [29], [34], [35], [38]. Another way to overcome the problem of phage resistance is to use therapeutic phage cocktails, which requires avoiding cross-resistance, i.e. bacterial mutants resistant to one phage must remain sensitive to the other components of the cocktail [29], [30], [48]. Thus, ideally, bacteriophages comprising the cocktail should employ different bacterial cell surface receptors [29], [48].

The purpose of this work was to study spontaneous and site-directed (non-polar) mutants of *Y. pestis* resistant to various bacteriophages, to identify phage receptors and to determine if phage-resistant mutants are impaired in virulence for mice. Nine phages capable of lysing *Y. pestis* were tested: L-413C, P2 *vir1*, ϕJA1, ϕA1122, Pokrovskaya, T7_Yp_, Y, PST, and R. We identified six receptors for eight bacteriophages in different parts of the LPS core. Altogether, the phages used at least seven different receptors in *Y. pestis*. Therefore, one can formulate a plague therapeutic cocktail containing several highly lytic phages exploiting different *Y. pestis* cell surface receptors. Most of the *Y. pestis* phage-resistant mutants were attenuated and thus should not present a problem for phage therapy. A direct correlation was observed between the length of the LPS core and virulence. The *yrbH* and *waaA* mutants were completely avirulent and thus LPS was shown to be a critical virulence factor of *Y. pestis*.

## Materials and Methods

### Ethics statement

Animal studies were conducted in compliance with the Animal Welfare Act and other federal, state and local laws and regulations regarding animal work, in a facility accredited by the Association for Assessment and Accreditation of Laboratory Animal Care International. All experiments with mice were performed under the animal use protocol approved by the Walter Reed Army Institute of Research Institutional Animal Care and Use Committee (Protocol #IB05-09).

### Bacteria, bacteriophages and growth media

Bacterial strains and bacteriophages used in this work are listed in Tables 1 and 2, respectively. High-concentration stocks of the ϕA1122, Pokrovskaya, Y, and T7_Yp_ phages were prepared using *Y. pestis* CO92 Pgm^−^ cultured at 28°C by the low multiplicity of infection method [66]. The same technique was used for large-scale isolation of the PST and R phages but they were grown on *Y. pseudotuberculosis* PB1. Finally, this method was used to propagate T7 on *E. coli* C600 at 37°C. The L-413C, ϕJA1, and P2 *vir1* phage stocks were prepared on *Y. pestis* CO92 Pgm^−^ as described previously [19] using different temperatures of incubation, 28°C for L-413C and ϕJA1 or 37°C for P2 *vir1*. Bacteria and bacteriophages were grown in liquid Brain Heart Infusion (BHI) medium (Becton-Dickinson, Franklin Lakes, New Jersey, USA) or on BHI plates containing 1.5% and 0.7% Bacto Agar (Becton-Dickinson) except *E. coli* strains, which were grown in LB broth (Becton-Dickinson) or on LB agar plates. The slants with tryptose blood agar (Becton-Dickinson) containing 5% defibrinated sheep blood (Lampire Biological Laboratories, Pipersville, Pennsylvania, USA) were used for streaking *Y. pestis* frozen stocks to prepare the challenge suspensions in animal experiments [67].

**Table 1:** Bacterial strains used in this work.

Strain	Relevant characteristics	Source/reference
***Yersinia pestis*** **:**		
CO92 Pgm^−^	Attenuated^a^, Pmx^r^ (polymyxin B resistant)	Lab collection [49]
CO92 Pgm^−^ *lpxM*	Km^r^-cassette replacement of YPO2063 (*lpxM*)^b^ gene, Pmx^r^	This work
CO92 Pgm^−^ *yrbH*	Km^r^-cassette replacement of YPO3577 (*yrbH*) gene, Pmx^s^	This work
CO92 Pgm^−^ *yrbH* (pBAD^c^)	Transformed with a pBAD vector (Ap^r^, Invitrogen)	This work
CO92 Pgm^−^ *yrbH* (pYrbH)	pYrbH is pBAD with cloned *yrbH*	This work
CO92 Pgm^−^ *waaA*	Km^r^-cassette replacement of YPO0055 (*waaA*) gene, Pmx^s^	This work
CO92 Pgm^−^ *waaA* (pBAD)	Transformed with pBAD	This work
CO92 Pgm^−^ *waaA* (pWaaA)	pWaaA is pBAD with cloned *waaA*	This work
CO92 Pgm^−^ *hldE*	Km^r^-cassette replacement of YPO0654 gene, Pmx^s^	This work
CO92 Pgm^−^ *hldE* (pBAD)	Transformed with pBAD	This work
CO92 Pgm^−^ *hldE* (pHldE)	P654 is pBAD with cloned YPO0654	This work
CO92 Pgm^−^ *waaF*	Km^r^-cassette replacement of YPO0057 (*waaF*) gene, Pmx^s^	This work
CO92 Pgm^−^ *waaF* (pBAD)	Transformed with pBAD	This work
CO92 Pgm^−^ *waaF* (pWaaF)	pWaaF is pBAD with cloned *waaF*	This work
CO92 Pgm^−^ *waaL*	Km^r^-cassette replacement of YPO0417 (*waaL*) gene, Pmx^r^	This work
CO92 Pgm^−^ *waaL* (pBAD)	Transformed with pBAD	This work
CO92 Pgm^−^ *waaL* (pWaaL)	pWaaL is pBAD with cloned *waaL*	This work
CO92 Pgm^−^ *wabD*	Km^r^-cassette replacement of YPO0187 gene, Pmx^r^	This work
CO92 Pgm^−^ *wabC*	Km^r^-cassette replacement of YPO0186 gene, Pmx^r^	This work
CO92	Fully virulent strain, from human case of pneumonic plague, Pmx^r^	Lab collection [52]
CO92 *yrbH-1*	Km^r^-cassette replacement of YPO3577 (*yrbH*) gene, Pmx^s^	This work
CO92 *yrbH-2*	An independent *yrbH* mutant, Pmx^s^	This work
CO92 *waaA-1*	Km^r^-cassette replacement of YPO0055 (*waaA*) gene, Pmx^s^	This work
CO92 *waaA-2*	An independent *waaA* mutant, Pmx^s^	This work
CO92 *hldE*	Km^r^-cassette replacement of YPO0654 gene, Pmx^s^	This work
CO92 *waaF*	Km^r^-cassette replacement of YPO0057 (*waaF*) gene, Pmx^s^	This work
CO92 *waaL*	Km^r^-cassette replacement of YPO0417 (*waaL*) gene, Pmx^r^	This work
CO92 S-2	Spontaneous L-413C-resistant mutant, Pmx^s^	This work
CO92 S-7	Spontaneous L-413C-resistant mutant, Pmx^r^	This work
KIM D27	KIM Pgm^−^, attenuated^a^, Pmx^r^	Lab collection [53]
A1122	Pgm^−^ pCD1^−^, attenuated^a^, Pmx^r^	Lab collection [54]
***Yersinia pseudotuberculosis*** **:**		
PB1	Serovar IB	Lab collection [55]
***Escherichia coli*** **:**		
C600	K-12 derivative; λ– *supE44 tonA21* (r_k_^+^m_k_^+^)	Lab collection [56]
C600 pUTKm	pUTKm^d^ is a Tn*903 kan* gene source [57]	Lab collection
TOP10	Δ*lacX74 deoR recA1 rpsL, endA1, nupG*	Invitrogen
C-520	“Restrictionless” P2 phage indicator strain	R. Calendar^e^ [58]

a These strains are attenuated due to a lack of pigmentation/siderophore yersiniabactin production genes and/or of pCD1 plasmid encoding type III secretion system and virulence Yop proteins (for review, see [1]).

b *Y. pestis* CO92 LPS gene designations are given according to [50], [51].

c Clones with a pBAD vector (Invitrogen, Carlsbad, California, USA) were used in *trans*-complementation experiments to exclude a possible impact of pBAD on LPS expression and phage susceptibility.

d Plasmid pUTKm was used as a source of kanamycin cassette.

e Department of Molecular and Cell Biology, University of California, Berkeley, California, USA.

**Table 2:** Bacteriophages used in this work.

Bacteriophage	Group	Source	Reference(s)	LPS residues critical for phage receptors (determined in this work)
L-413C	P2	Lab collection	[14], [19], [23], [24], [27]	GlcNAc
P2 *vir1*	P2	R. Calendar	[59]	GlcNAc
ϕJA1^a^	P2?	Sewage	This work	Kdo/Ko
ϕA1122	T7	M.E. Schriefer^b^	[10], [18], [22], [25]–[27]	Kdo/Ko
T7	T7	I.J. Molineux^c^	[60]	NA^d^
T7_Yp_ e	T7	T7 host range mutant	This work	HepI/Glc
Pokrovskaya	ND^f^	B.B. Atshabar^g^	[9], [14], [23], [24]	HepII/HepIII
Y	T3?	ATCC	[12], [13]	HepI/Glc
PST	T2?	ATCC	[12], [61]–[63]	HepII/HepIII
R^h^	ND	ATCC	[12], [15], [63]–[65]	Beyond LPS core

a Phage ϕJA1 isolated from sewage lyses multiple *Y. pestis* strains but not *Y. pseudotuberculosis* or *E. coli* K-12; it has some degree of homology to L-413C because its DNA is amplified in qPCR with one of two tested pairs of primers specific for L-413C (A.A. Filippov, Y. He, and K.V. Sergueev, unpublished data).

b Bacterial Diseases Branch, Division of Vector-Borne Infectious Disease, National Center for Zoonotic, Vector-Borne and Enteric Diseases, Centers for Disease Control and Prevention, Ft. Collins, Colorado, USA.

c Section of Molecular Genetics and Microbiology, and Institute for Cellular and Molecular Biology, University of Texas at Austin, Austin, Texas, USA.

d NA, not applicable.

e T7_Yp_ is a T7 host range mutant isolated on the lawn of *Y. pestis* CO92; T7_Yp_ produces plaques on *Y. pestis* but not on *Y. pseudotuberculosis* or *Yersinia enterocolitica* and has a low plaquing efficiency on *E. coli* K-12 (A.A. Filippov, Y. He, and K.V. Sergueev, unpublished data).

f ND, not determined.

g Kazakh Scientific Center for Quarantine and Zoonotic Diseases, Almaty, Kazakhstan.

h R phage (R stands for “Russian” [12]) was originally isolated and studied by R.I. Kotlyarova [64] and is also designated as the Kotlyarova phage. Pseudotuberculosis diagnostic phages PST [63] and R [15] can lyse 92–100% of *Y. pseudotuberculosis* strains but are also active against the majority of *Y. pestis* strains.

### Bacteriophage assays

SM buffer [66] was used for bacteriophage storage and dilutions. Phage plaque assays were performed by the double-layer agar method as described earlier [66] with overnight incubation for L-413C, P2 *vir1*, ϕJA1, Y, PST, and R, or 5–6 h incubation for ϕA1122, Pokrovskaya, T7, and T7_Yp_. Plaquing (plating) efficiency tests were done as described previously [19] using an incubator temperature of 28°C. Phage adsorption experiments were performed according to [19].

### Isolation of spontaneous phage-resistant mutants and determination of the mutation frequencies

Spontaneous *Y. pestis* mutants resistant to bacteriophages were isolated from avirulent strains CO92 Pgm^−^, KIM D27, and A1122, as well as from wild-type virulent CO92 by plating on double-layer agar with the phage, 10^9^ PFU (plaque-forming unit) per plate. To determine the mutation frequencies per cell per generation, ten isolated colonies were picked out from each strain, inoculated into ten tubes with BHI, grown with agitation until approximate concentration of 10^9^ CFU/ml (colony-forming unit) and diluted in ten-fold increments down to 10^−7^. 0.1-ml aliquots from the 10^0^–10^−3^ dilutions were inoculated onto the double-layer plates and were incubated at 28°C for six days because some of the phage-resistant mutants grow very slowly. Simultaneously, 0.1-ml aliquots of 10^−5^, 10^−6^, and 10^−7^ dilutions were plated on BHI agar without phage (three plates for each dilution), for determination of CFU numbers. To calculate mutation frequencies, mean numbers of mutants in one ml of culture were divided by mean total numbers of live bacterial cells. The phage resistant colonies were picked up and purified by two passages on double-layer plates with the phage and were used in further experiments.

### Site-directed mutagenesis

Site-directed mutagenesis of *Y. pestis* CO92 LPS genes was performed by one-step PCR-mediated in-frame replacement with the Tn*903* kanamycin cassette as described earlier [68]. This kanamycin resistance gene, *aph(3′)-Ia* [69], does not provide cross-resistance to streptomycin and gentamicin [70]; the procedure was approved by the WRAIR Institutional Biosafety Committee (the protocol MUA #125-a, June 14, 2004) and by CDC (Memorandum of February 25, 2008). Primers used for mutagenesis and verifying the correctness of replacement were designed using NetPrimer online program (Premier Biosoft International, http://www.premierbiosoft.com) and are listed in Table S1. The mutagenesis primers were designed so that the replacements were non-polar and preserved adjacent genes including ribosome-binding sites intact.

### Testing potential phenotypic alterations in *Y. pestis* phage-resistant mutants

Spontaneous and induced *Y. pestis* phage-resistant mutants were tested for plasmid content by the method of Kado and Liu [71], for pigmentation by plating on Congo red agar [72], for low calcium response on magnesium-oxalate agar plates [73], and for possible loss of natural polymyxin resistance associated with rough type of LPS on BHI agar with polymyxin B (10 and 150 U/ml; Sigma-Aldrich, St. Louis, Missouri, USA) and without the antibiotic.

### Molecular cloning and *trans*-complementation

*Y. pestis* LPS biosynthesis genes were cloned into a pBAD-TOPO vector using pBAD TOPO® TA Expression Kit (Invitrogen) according to recommendations of the supplier. Primers for PCR cloning are presented in Table S2. All genes were cloned under the control of pBAD arabinose promoter, except *Y. pestis yrbH*, which was cloned under the control of its own promoter, as described elsewhere [74]. Two tandem stop codons (see Table S2) were placed immediately upstream of the *yrbH* promoter to prevent the expression of a truncated protein from the pBAD arabinose promoter. The knockout mutants were cured of a mutagenesis helper plasmid pKD46 [68] by growing at increased temperature, at 38°C. The recombinant plasmids were introduced into corresponding mutants using electroporation. All mutants were also transformed with DNA of the intact pBAD (to exclude a possible impact of the vector plasmid on LPS expression and phage susceptibility). Transformants that acquired a recombinant plasmid or the vector without an insert were tested for plaquing efficiencies on the double-layer BHI plates supplemented with 10 mM of L-arabinose to induce the pBAD promoter, as well as for the LPS size (see below).

### SDS-PAGE analysis of LPS

The presence and size of LPS core in *Y. pestis* strains was analyzed as described previously [74], with the following minor modifications. *Y. pestis* strains were grown overnight at 28°C in BHI. After the lysis and deproteinization, LPS was visualized by fluorescent staining using Pro-Q® Emerald 300 Lipopolysaccharide Gel Stain Kit (Invitrogen).

### Infection of mice

*Y. pestis* strains for animal challenge experiments were isolated from single colonies, tested for the presence of three virulence plasmids [71], pigmentation [72], and low calcium response [73] and kept at −80°C in BHI with 10% dimethyl sulfoxide. The challenge culture suspensions were prepared from the frozen stocks according to [67] but using normal saline instead of HIB broth. Mouse lethality was determined following subcutaneous inoculation of 100 µl saline containing each of various 10-fold concentrations of *Y. pestis* into groups of ten 8-week-old female BALB/c mice. The mice were observed twice a day until 14 days postinfection. Median lethal doses (LD_50_) were calculated by the method of Reed and Muench [75]. In addition to the number of deaths, the mean time to death (MTD) was recorded.

## Results

### Spontaneous bacteriophage resistance mutations in *Y. pestis*

First, we determined the frequencies of spontaneous mutations resulting in resistance of *Y. pestis* towards four different phages, L-413C, ϕA1122, Pokrovskaya, and Y (Table 3). *Y. pestis* CO92 showed different mutation frequencies to the L-413C, Pokrovskaya, and Y phages. No ϕA1122-resistant mutations were observed. Six independent mutants resistant to L-413C (two for each of the three strains, CO92 Pgm^−^, KIM D27, and A1122) were isolated and their phenotypes tested. Five out of six clones lost the native resistance to polymyxin B associated with the rough form of *Y. pestis* LPS, suggesting LPS core damage [51], [76]. Since some phage-resistant mutants of *Y. pestis* previously showed pleiotropic effects on several phenotypic traits [77], [78], we tested L-413C-resistant mutants for plasmid content, pigmentation, and low calcium response and observed no alterations in these traits in comparison with the parental strains. All six mutants were found to remain sensitive to ϕA1122.

**Table 3:** Frequencies of *Y. pestis* spontaneous mutations to bacteriophage resistance.

*Y. pestis* strain	Mutation frequency of resistance to:			
	L-413C	ϕA1122	Pokrovskaya	Y
CO92 Pgm^−^	1.2×10^−4^	<3.1×10^−10^	5.5×10^−6^	5.3×10^−7^
KIM D27	1.1×10^−4^	<4.2×10^−10^	NT^a^	NT
A1122	1.0×10^−4^	NT	NT	NT

a NT, not tested.

### Identification of bacteriophage receptors

**Figure 1:**
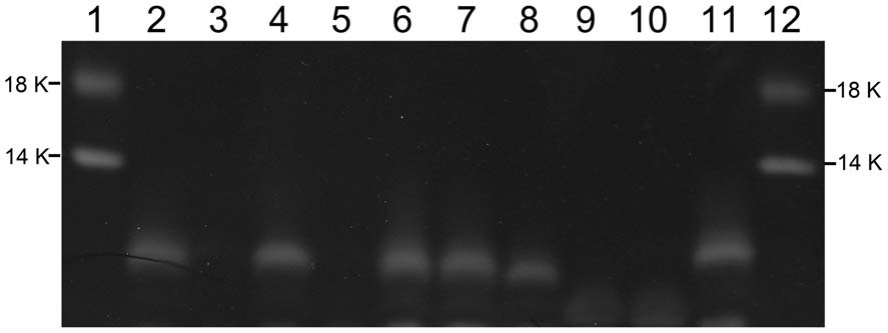
LPS defects in *Y. pestis* knockout mutants. 20 µl of deproteinized lysates [74] were loaded on 14% SDS-PAGE, run and stained with Pro-Q® Emerald 300 Lipopolysaccharide Gel Stain Kit (Invitrogen). 1 and 12, CandyCane™ Glycoprotein Molecular Weight Standards (Invitrogen); 2, CO92 Pgm^−^; 3, CO92 Pgm^−^ *yrbH*, 4, CO92 Pgm^−^ *lpxM*; 5, CO92 Pgm^−^ *waaA*, 6, CO92 Pgm^−^ *wabC*; 7, CO92 Pgm^−^ *wabD*; 8, CO92 Pgm^−^ *waaL*; 9, CO92 Pgm^−^ *waaF*; 10, CO92 Pgm^−^ *hldE*; 11, CO92 Pgm^−^ *yrbH* (pYrbH).

To identify bacteriophage receptors, a series of defined non-polar *Y. pestis* CO92 Pgm^−^ mutants affected in genes encoding the synthesis of different parts of LPS (Table 1) were obtained by one-step knockout kanamycin cassette mutagenesis [68]. Several LPS biosynthesis genes were cloned in a pBAD vector (Invitrogen) and used for *trans*-complementation tests. Both knockout mutants and *trans*-complemented clones were tested for susceptibility to nine bacteriophages using comparative plaquing efficiency assays (see Tables 4 and 5). Such approach has been recently successfully used for identification of T7 receptor in *E. coli* K-12 [79]. We additionally tested the knockout mutants for the efficiency of phage adsorption in comparison with the parental strain CO92 Pgm^−^ (Table 6). The results of mutagenesis were verified by PCR (data not shown) and fluorescent staining of LPS that demonstrated an increase in electrophoretic mobility or the loss of LPS bands in the mutants (Fig. 1).

**Table 4:** Plaquing efficiencies of various phages on *Y. pestis* CO92 LPS-affected mutants.

*Y. pestis* strain^a^	Efficiencies of plating^b^								
	L-413C	P2 *vir1*	ϕJA1	ϕA1122	Pokrovskaya	T7_Yp_	Y	PST	R
CO92 Pgm^−^ (parental)	1.0	1.0	1.0	1.0	1.0	1.0	1.0	1.0	1.0
*lpxM*	1.1	0.8	0.1	0.4	1.2	1.7	1.0	1.1	1.0
*yrbH*	<2.0×10^−12^	<5.0×10^−8^	<7.1×10^−10^	1.2×10^−4^	6.9×10^−12^	1.1×10^−9^	5.3×10^−9^	<7.1×10^−12^	0.1
*waaA*	<2.0×10^−12^	<5.0×10^−8^	<7.1×10^−10^	1.4×10^−3^	<7.7×10^−13^	1.3×10^−7^	3.0×10^−7^	<7.1×10^−12^	20
*hldE*	<2.0×10^−12^	<5.0×10^−8^	7.1×10^−5^	0.8	1.1×10^−3^	3.3×10^−2^	5.6×10^−6^	<7.1×10^−12^	120
*waaF*	2.8×10^−4^	<5.0×10^−8^	3.6×10^−5^	0.6	8.5×10^−4^	0.1	0.2	<7.1×10^−12^	60
*waaL*	<2.0×10^−12^	<5.0×10^−8^	6.4×10^−3^	0.9	0.8	1.9	1.0	1.4	300
*wabD*	0.9	0.7	1.9	1.2	1.4	1.8	0.7	1.2	90
*wabC*	1.0	1.0	3.5	1.4	2.0	0.7	1.3	0.9	210

a The phage plaquing tests were performed at 28°C.

b The titers of bacteriophages (PFU/ml) determined on CO92 Pgm^−^ strain were: 5.0×10^11^ (L-413C), 2.0×10^7^ (P2 *vir1*), 1.4×10^9^ (ϕJA1), 5.0×10^12^ (ϕA1122), 1.3×10^12^ (Pokrovskaya), 9.0×10^11^ (T7_Yp_), 3.0×10^11^ (Y), 1.4×10^11^ (PST), and 1.0×10^9^ (R).

**Table 5:** Restoration of phage susceptibility as a result of *trans*-complementation of *Y. pestis* LPS-affected mutants.

*Y. pestis* strain^a^	Susceptibility to^b^:							
	L-413C	P2 *vir1*	ϕJA1	ϕA1122	Pokrovskaya	T7_Yp_	Y	PST
CO92 Pgm^−^ (parental)	**+**	**+**	**+**	**+**	**+**	**+**	**+**	**+**
*yrbH* c	**−**	**−**	**−**	**−**	**−**	**−**	**−**	**−**
*yrbH* (pYrbH^d^)	**+**	**+**	**+**	**+**	**+**	**+**	**+**	**+**
*waaA*	**−**	**−**	**−**	**−**	**−**	**−**	**−**	**−**
*waaA* (pWaaA)	**+**	**+**	**+**	**+**	**+**	**+**	**+**	**+**
*hldE*	**−**	**−**	**−**	**+**	**−**	**−**	**−**	**−**
*hldE*(pHldE)	**+**	**+**	**+**	**+**	**+**	**+**	**+**	**+**
*waaF*	**−**	**−**	**−**	**+**	**−**	**+**	**+**	**−**
*waaF* (pWaaF)	**+**	**+**	**+**	**+**	**+**	**+**	**+**	**+**
*waaL*	**−**	**−**	**−**	**+**	**+**	**+**	**+**	**+**
*waaL* (pWaaL)	**+**	**+**	**+**	**+**	**+**	**+**	**+**	**+**

a The phage plaquing tests were performed at 28°C.

b **+**, phage-susceptible; **−**, phage-resistant.

c Each of the mutants was also transformed with a pBAD vector without insert to exclude possible impact of the vector on LPS structure and phage susceptibility; all pBAD^+^ transformants had the same phenotype as the parental mutant (data not shown).

d See characteristics of the recombinant plasmids in Table 1 and Materials and Methods.

**Table 6:** Phage adsorption tests on *Y. pestis* LPS mutants.

*Y. pestis* strain	Average percentage of remaining phage particles (±SD)						
	L-413C	P2 *vir1*	ϕA1122	Pokrovskaya	T7_Yp_	Y	R
None (control)	100%	100%	100%	100%	100%	100%	100%
*yrbH*	100%*	100%*	78.0%(±4.9)	100%*	93.2%(±6.8)	100%*	44.9% (±6.5)
*hldE*	96.2%(±3.7)	92.3%(±13.3)	29.3%(±2.4)	98.9%(±2.1)	82.5%(±9.9)	60.5%(±4.5)	42.7% (±4.0)
*waaF*	57.3%(±8.2)	22.0%(±1.5)	9.5%(±1.0)	82.1%(±6.3)	62.5% (±5.3)	43.0%(±6.0)	36.8% (±4.6)
*waaL*	63.4%(±9.1)	23.1%(±2.3)	6.1%(±1.2)	12.9%(±2.3)	35.0% (±2.5)	20.3%(±0.8)	35.0% (±1.9)
*wabC*	1.1%(±0.2)	0%	6.3%(±0.1)	14.4%(±3.0)	35.9% (±4.2)	10.1%(±2.5)	38.5% (±4.3)
CO92 (parental)	1.3%(±0.3)	0.2%(±0.1)	6.7%(±0.6)	13.2%(±2.7)	34.2% (±5.1)	15.2%(±3.4)	38.8% (±4.2)

*Percentages were rounded to 100% when the number of estimated remaining phage was greater than that remaining in the control.

The fine structure of LPS in all kinds of *Y. pestis* knockout mutants used in our work has been previously characterized in details using mass spectroscopy analysis in strains KIM6 of bv. Medievalis [80] (for the *waaA* and *yrbH* mutants) and 231 of bv. Antiqua [51] (for the rest of mutants). We used BLAST analysis [81] for comparison of 11 LPS biosynthesis proteins (YrbH, WaaA, HldE, WaaF, WaaL, WabC, WabD, WecA, WaaQ, WaaE, and WaaC) including all those, the genes for which were knocked out in this work, in 23 published whole genome sequences of *Y. pestis* strains of various biovars deposited in the NCBI databases (http://www.ncbi.nlm.nih.gov/). They showed 100% identity, or, in rare cases, 99% identity of amino acid sequences. This suggested that the LPS core structure of diverse *Y. pestis* strains is highly conserved and thus we could use the structural data of other authors [51], [80].

**Figure 2:**
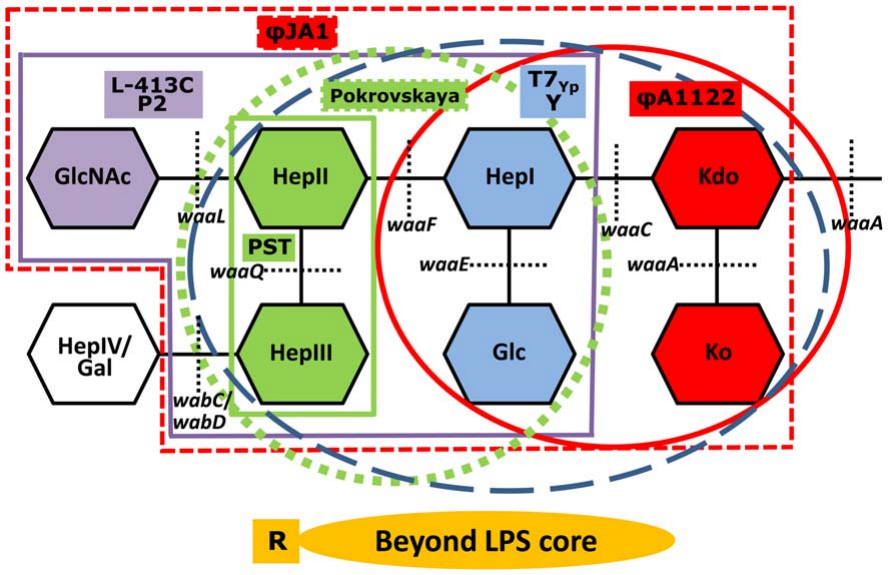
Identification of *Y. pestis* cell surface receptors for different bacteriophages. The structure of LPS core is presented according to [51]. Kdo, 2-*keto*-3-deoxy-octulosonic acid; Ko, d-*glycero*-d-*talo*-oct-2-ulosonic acid; Hep, heptulose (ketoheptose); Glc, glucose; Gal, galactose; GlcNAc, N-acetylglucosamine. Black dashed lines designate the sites where the gene product, a corresponding glycosyltransferase, forms a glycosidic bond. The *yrbH* mutations affecting Kdo synthesis have the same effect on the LPS structure as *waaA* mutations, and defect in the *hldE* gene involved in ADP-l,d-heptose synthesis causes the same phenotype as the *waaC* mutation [51]. The critical sugar residues for certain phage receptors have solid color fill matching with that of phage designations and with the color of lines outlining the receptors. The phage receptors are outlined with bold purple (for the L-413C and P2 phages), dashed red (for ϕJA1), bold green (for PST), dashed green (for Pokrovskaya), dashed blue (for T7_Yp_ and Y), and bold red (for ϕA1122) lines.

In contrast to the data of another group based on multi-step suicide plasmid mutagenesis [78], [82], in our experiments *lpxM* (*msbB*) mutants with altered lipid A acylation were susceptible to L-413C, as well as to eight other phages (Table 4). Mutations in genes responsible for early steps of LPS core synthesis, *yrbH* and *waaA*, resulted in the loss of both outer and inner LPS core [80] (see Fig. 2). The coreless mutants had slower growth [80], especially in early generations, and displayed resistance to eight phages of the nine tested (Table 4), suggesting that the receptors for these eight bacteriophages lie in the LPS core. Mutants affected in carbohydrate residues of the main LPS core chain (*hldE*, *waaF*, and *waaL*) were completely resistant to the L-413C and P2 *vir1* phages (Table 4 and Fig. 2). The *waaL* mutants are deficient in O antigen ligase, but in *Y. pestis* lacking O antigen the WaaL protein forms a glycosidic bond between l-*glycero*-d-*manno*-heptopyranose (HepII) and a terminal outer core residue, β-*N*-acetyl-d-glucosamine (GlcNAc) [51]. Since *waaL* mutants carried the minimal LPS outer core defect (lack of GlcNAc) and still lost phage susceptibility, we concluded that a critical part of the receptor for L-413C and P2 *vir1* is the terminal GlcNAc residue of the LPS outer core (Fig. 2). This was confirmed by *trans*-complementation of *waaL* mutation with the *waaL* gene cloned in a pBAD vector, which resulted in full restoration of susceptibility to L-413C and P2 *vir1* (Fig. S1 and Table 5). Adsorption tests (Table 6) showed that not only *waaL* but also *waaF* mutant was affected in the phage binding, and *hldE* mutant completely lost the ability to adsorb to L-413C and P2 *vir1*. Thus HepII/HepIII and HepI/Glc residues are also involved in the formation of L-413C and P2 *vir1* receptors (Fig. 2; the receptor is outlined with bold purple line).

The *waaL* mutation resulted in reduction of the ϕJA1 phage efficiency of plating by more than two logs (Table 4) suggesting that GlcNAc is involved in ϕJA1 reception (Fig. 2). Both *waaF* and *hldE* mutants further decreased plaquing efficiency by two more orders of magnitude (Table 4) and thus HepII/HepIII residues also take part in the formation of ϕJA1 receptor. Finally, ϕJA1 did not form plaques on the coreless mutants *yrbH* and *waaA* providing at least five log difference in the efficiencies of plating in comparison with the *waaF* and *hldE* mutants. This suggests that the ϕJA1 phage receptor includes both LPS outer and inner core sugars and Kdo/Ko residues form a critical part of the receptor (Fig. 2).

Using the same approach (see Tables 4, 5, and 6), we localized five more receptors for six other phages (Table 2 and Fig. 2). Since WaaA forms both GlcN-Kdo and Kdo-Ko bonds and we did not mutagenize *waaE* and *waaQ* genes, we could not identify a single sugar residue most critical for phage binding in these cases but mapped most important receptor components to three different pairs of adjacent inner core residues, Kdo/Ko (for ϕA1122 and ϕJA1), HepI/Glc (for T7_Yp_ and Y), and HepII/HepIII (for Pokrovskaya and PST). This was confirmed by *trans*-complementation tests with recombinant plasmids carrying the *yrbH*, *waaA*, *hldE*, and *waaF* genes (Table 5). The restoration of LPS core with normal mobility was shown in the complemented mutants (see Fig. 1).

The adsorption assays (Table 6) confirmed the data of plating efficiency tests on the most important receptor components and revealed some additional residues involved in the formation of phage receptors. For example, the experiments with the ϕA1122 phage confirmed that Kdo/Ko residues are a critical part of the receptor: the *yrbH* mutant lost 78% of its phage binding activity. Additionally, the *hldE* mutant showed some decreased ϕA1122 adsorption, suggesting that HepI/Glc residues are also involved in the phage reception. The adsorption experiments also allowed us to expand the receptors for the Pokrovskaya, T7_Yp_ and Y phages (Table 6 and Fig. 2). The adsorption tests with the ϕJA1 and PST phages failed; we could not determine any binding even to *Y. pestis* CO92 Pgm^−^ strain supposed to contain the intact bacteriophage receptors. The cause could be a low efficiency binding resulting in disruption of the phage-bacterium bonds when starting centrifugation of the bacterial suspensions infected with these two phages. Our data showed that the receptor for the R phage is located beyond the LPS core (see Tables 4 and 6 and Fig. 2).

### Attenuation of *Y. pestis* phage-resistant mutants in mice

To determine if bacteriophage resistance mutations impact on *Y. pestis* virulence, we reproduced site-directed mutagenesis [68] of five genes (*waaL*, *waaF*, *hldE*, *waaA*, and *yrbH*) in the fully virulent wild type strain CO92 (Pgm^+^) (Table 1). Additionally, eight spontaneous mutants of CO92 resistant to the L-413C phage were isolated. All remained susceptible to ϕA1122. Seven of them became sensitive to polymyxin B. One Pmx^s^ (S-2) and one Pmx^r^ (S-7) spontaneous mutants were selected for further work. LD_50_ doses were determined for the two spontaneous and five site-directed phage-resistant mutants in comparison with the parental strain CO92 in BALB/c mice after subcutaneous injection (see Table 7). Six phage-resistant mutants of seven tested (86%) were shown to be attenuated, with significantly higher LD_50_ and longer mean times to death (MTD). Three of them (S-2, *yrbH-1*, and *waaA-1*) completely lost virulence. Only one spontaneous mutant resistant to L-413C (S-7) was fully virulent. Both *yrbH* and *waaA* mutants lack the LPS outer and inner core ([80]; see also Fig. 1, lanes 3 and 5) and they both became fully avirulent. The same results were obtained in a repeated experiment with independently derived mutants, *yrbH-2* and *waaA-2* (Table 7).

**Table 7:** Attenuation of spontaneous L-413C-resistant and defined LPS-affected mutants for BALB/c mice infected subcutaneously.

Strain	LD_50_ (CFU)	Attenuation (fold)	Mean time to death (days)	MTD extension
CO92 (WT)	3.3	―	5.5	―
S-2	≥9.8×10^7^	≥3.0×10^7^	>14.0	>150%
S-7	1.7	―	5.7	―
*waaL*	44.4	13.5	5.8	―
*waaF*	4.5×10^2^	1.4×10^2^	9.0	64%
*hldE*	6.3×10^3^	1.9×10^3^	11.9	116%
*waaA-1*	≥1.3×10^7^	≥3.9×10^6^	>14.0	>150%
*yrbH-1*	≥3.4×10^7^	≥1.0×10^7^	>14.0	>150%
CO92 (WT)*	2.2	―	5.4	―
*waaA-2* *	≥8.0×10^6^	≥3.6×10^6^	>14.0	>150%
*yrbH-2* *	≥9.1×10^6^	≥2.8×10^6^	>14.0	>150%

*Repeated experiment.

## Discussion

The emergence of multidrug-resistant strains of *Y. pestis* [4] may result in outbreaks of untreatable bubonic and pneumonic plague with very high mortality and suggests the paramount importance of finding adequate alternatives to antibiotics including bacteriophages [5], which have already shown a high efficacy for the therapy of several other infections [6]–[8]. One of the major problems that can arise when using phages as therapeutics is the development of phage-resistant mutants [28]–[30], most frequently resulting from the alteration or loss of the bacterial cell surface receptor [31]–[33]. To solve this problem, one can use therapeutic cocktails consisting of phages that exploit different bacterial receptors [29], [30], [48]. Another way is to use the phages adsorbing on pathogenicity factors so that the receptor mutations resulting in phage resistance simultaneously attenuate the bacterium, which is subsequently eliminated from the host by the immune system [29], [34], [35], [38]. Given the high potential of bacteriophages as alternative therapies against drug-resistant plague, here we addressed the problem of phage-resistant mutants in *Y. pestis* and both strategies of its overcoming: identified *Y. pestis* receptors for a number of bacteriophages and determined if the phage-resistant mutants become attenuated.

Nine phages able to lyse *Y. pestis* were tested. They included four plague diagnostic phages, L-413C [14], [19], [23], [24], [27], ϕA1122 [10], [18], [22], [25]–[27], Pokrovskaya [9], [14], [23], [24], and Y [12], [13]; two pseudotuberculosis diagnostic phages also capable of lysing most *Y. pestis* strains, PST [12], [61]–[63] and R [12], [15], [63]–[65]; as well as P2 *vir1*, a virulent mutant of well-known coliphage P2 [59] previously shown to lyse *Y. pestis* at 37°C [19], [27]. Additionally, we isolated a new *Y. pestis*-specific phage from sewage, ϕJA1, and also selected a host range mutant of enterobacteriophage T7, T7_Yp_, which showed both a striking specificity and high lytic activity towards *Y. pestis*. First, we demonstrated a significant difference in the frequencies of spontaneous mutations of *Y. pestis* resistance to phages belonging to different morphologic and taxonomic groups. For example, resistance to L-413C was a rather frequent event (about 10^−4^ per cell per generation), whereas we could not isolate any spontaneous mutants resistant to ϕA1122, suggesting that these two phages use different bacterial surface receptors for their adsorption. Most L-413C-resistant clones became sensitive to polymyxin B, indicating that they have an LPS core defect [51], [76]. All mutants resistant to L-413C remained sensitive to ϕA1122. This finding also suggested that L-413C and ϕA1122 exploit different surface structures as the receptors.

Site-directed mutagenesis of different LPS genes, mainly those encoding for glycosyltransferases involved in the synthesis of the LPS outer and inner core, and *trans-*complementation with the cloned genes followed by efficiency of plating and adsorption tests (Tables 4, 5, 6) allowed us to localize six *Y. pestis* cell surface receptors for bacteriophages in different parts of the LPS core (Fig. 2). It was demonstrated that the terminal residue of the outer core, N-acetylglucosamine, is a critical part of L-413C and P2 *vir1* receptor. The most important parts of the rest of the receptors were mapped to three different pairs of adjacent *Y. pestis* LPS inner core residues, Kdo/Ko (for the ϕA1122 and ϕJA1 phages), HepI/Glc (for T7_Yp_ and Y), and HepII/HepIII (for Pokrovskaya and PST). Apart from the critical receptor components, we found some additional LPS sugar residues involved in the phage adsorption that made up six different receptor structures for eight phages (Fig. 2). The seventh receptor, for the R phage, was shown to lie beyond the LPS core.

The location of phage receptors in different LPS core residues can at least partially explain the difference in the frequencies of phage resistance mutations. For example, L-413C provided the highest frequency of resistance, most probably due to the largest number of genes, mutations in which result in the receptor loss (*waaL*, *wecA*, *waaF*, *waaC*, *hldE*, *waaA*, *yrbH* and other genes responsible for Kdo biosynthesis; see Fig. 2, Refs. 51 and 80). At the same time, ϕA1122 binds to Kdo and HepI/Glc residues that can be affected by mutations in a relatively small number of genes.

Therefore, nine bacteriophages tested in this work can use at least seven different receptors in *Y. pestis*. Based on the principle of formulating optimal therapeutic cocktails from the phages that employ different cell surface receptors [29], [30], [48] and on our results, we recommend six bacteriophages as the best candidates for a plague therapeutic cocktail: ϕA1122, Pokrovskaya, Y, T7_Yp_, ϕJA1, and R. They use different receptors in *Y. pestis*. The best candidate in this list is the ϕA1122 phage previously shown to be rather specific [22], [25]–[27], highly lytic [27] and active against virtually all known *Y. pestis* isolates [18], [22]. We identified the *Y. pestis* receptor for ϕA1122 and demonstrated that the mutation of resistance to this phage is a very rare event. There are only two known *Y. pestis* strains resistant to ϕA1122 out of thousands tested [18]. It is also important that ϕA1122 genome has been sequenced and no potentially detrimental genes were found in it [18]. The Pokrovskaya [14], [23], [24], Y [12], [13], and R [15], [63], [65] phages have been shown to be highly active against *Y. pestis* (especially, Pokrovskaya and Y) and to have broad ranges of susceptible strains. Our results provided important new information about the receptors for these bacteriophages, making them also promising as potential components of plague therapeutic cocktails. Finally, two new phage isolates, T7_Yp_ (a coliphage T7 host range mutant) and ϕJA1 (obtained from sewage) demonstrate a high specificity and significant lytic potential towards *Y. pestis*, particularly, T7_Yp_ (A.A. Filippov, Y. He, and K.V. Sergueev, unpublished data) and use known receptors on the bacterial cell surface, which also makes them good candidates for future plague therapeutic cocktails. For T7_Yp_, it is also important that its parental phage, T7, has been sequenced and no genes potentially harmful for warm-blooded animals were identified [83].

Determination of median lethal doses in BALB/c mice by subcutaneous route of administration for two spontaneous *Y. pestis* L-413C-resistant mutants and seven defined LPS-affected mutants resistant to different phages showed that eight of them (89%) were attenuated, displaying a significant increase in LD_50_ and MTD (Table 7). Five mutants (S-2, *waaA-1*, *waaA-2*, *yrbH-1*, and *yrbH-2*) became completely avirulent. Only one spontaneous mutant resistant to L-413C (S-7) that retained polymyxin resistance was fully virulent. This mutation seems to be unrelated to the phage receptor, instead affecting some steps of phage lytic cycle following its adsorption, e.g., DNA replication or phage assembly [31]. The attenuation of most phage-resistant strains suggests that the emergence of such mutants *in vivo* should result in their elimination by the immune system and thus should not significantly decrease the efficiency of phage therapy.

Site-directed mutations in each of the three tested genes encoding for glycosyltransferases that incorporate different sugar residues into the LPS core (*waaA*, *waaF*, and *waaL*), as well as in two genes essential for Kdo (*yrbH*) and ADP-l,d-heptose (*hldE*) biosynthesis were shown to affect *Y. pestis* virulence (Table 7). It has been previously found that mutants of *Yersinia enterocolitica* [84], *Salmonella enterica* [85], *Actinobacillus pleuropneumoniae* [86], and *Burkholderia cenocepacia* [87] with different LPS core defects demonstrate reduced virulence. In *Y. pestis*, *in vivo* maintenance of a tetra-acylated structure of lipid A in LPS has been shown to be essential for mouse virulence after subcutaneous infection [88]. Recently, five LPS core mutants of *Y. pestis* 231 (biovar Antiqua) have been tested for virulence in mice and guinea-pigs challenged subcutaneously, *wabD, waaL, waaQ, waaE, and hldE* [51]. Of them, *waaL* and *waaE* mutants showed a slight increase in LD_50_ for mice and guinea pigs, whereas *hldE* was attenuated by four logs. Our results on the *waaL* and *hldE* mutants are in agreement with the previous data [51]. Additionally, our data (Table 7) show that there is a clear correlation between the degree of LPS core truncation and attenuation. Some spontaneous undefined *Y. pestis* mutants resistant to the Pokrovskaya and/or L-413C phages have been observed to have reduced virulence or to become avirulent for mice or guinea pigs [77]. Here we showed that the most likely cause of attenuation of such mutants is the LPS core truncation.

It is important that both *yrbH* and *waaA* mutants (two pairs of independently derived clones) lacking the LPS core [74], [80] demonstrated full avirulence (Table 7). These two types of mutants have different mechanisms of the loss of LPS core. The *waaA* product is a transferase catalyzing Kdo glycosylation of lipid A [80], while the YrbH protein is arabinose 5-phosphate isomerase that converts ribulose 5-phosphate into arabinose 5-phosphate, which is the first committed step in the Kdo biosynthesis [74], [80]. The complete loss of virulence in the two types of LPS coreless mutants suggests that LPS is a critical virulence factor of *Y. pestis*. These results are of great importance for the molecular pathogenesis of plague because previously only the loss of major components of type III secretion system encoded on the plasmid pCD1 [89]–[91], a chromosomal gene cluster of siderophore yersiniabactin synthesis and reception [91]–[94] or a gene encoding for the NlpD lipoprotein [95] have been known to completely attenuate *Y. pestis* strains.

## Supporting Information

Figure S1 ***Trans*** **-complementation of L-413C phage receptor defect.** A, B, and C: L-413C plaquing on *Y. pestis* CO92 Pgm^−^, CO92 Pgm^−^ *waaL*, and CO92 Pgm^−^ *waaL* (pWaaL), respectively.
(TIF)

Table S1 Primers for mutagenesis of *Y. pestis* LPS genes and verifying the sizes of amplicons. Notes: ^a^50-bp flanking sequences of *Y. pestis* chromosomal DNA providing site-specific insertion of kanamycin cassette are shown in lower case type. ^b^F1 and R1 primers targeted *Y. pestis* DNA on the gene flanks and provided amplification of a fragment with changed size due to the replacement with the Km^r^ gene. ^c^Int (internal) primers amplified the corresponding intact *Y. pestis* gene and were used to exclude gene duplications and emergence of merodiploids. ^d^The *kan*-start and *kan*-stop primers targeted the Km^r^ gene and were used to amplify the DNA novel joints with corresponding F1 and R1 primers, respectively.
(DOCX)

Table S2 Primers for cloning of *Y. pestis* LPS genes. Notes: ^a^Two tandem stop codons in the *yrbH* forward primer are underscored. Lower case letters in the rest of forward primers designate modified ribosome binding site according to recommendations of the supplier of the TOPO cloning system (Invitrogen).
(DOCX)
