# Supplementary figures and images for: Bacteriophage-Resistant Mutants in Yersinia pestis: Identification of Phage Receptors and Attenuation for Mice
Source: PLoS One. 2011 Sep 28;6(9):e25486. doi: 10.1371/journal.pone.0025486 (PMC3182234; doi:10.1371/journal.pone.0025486)

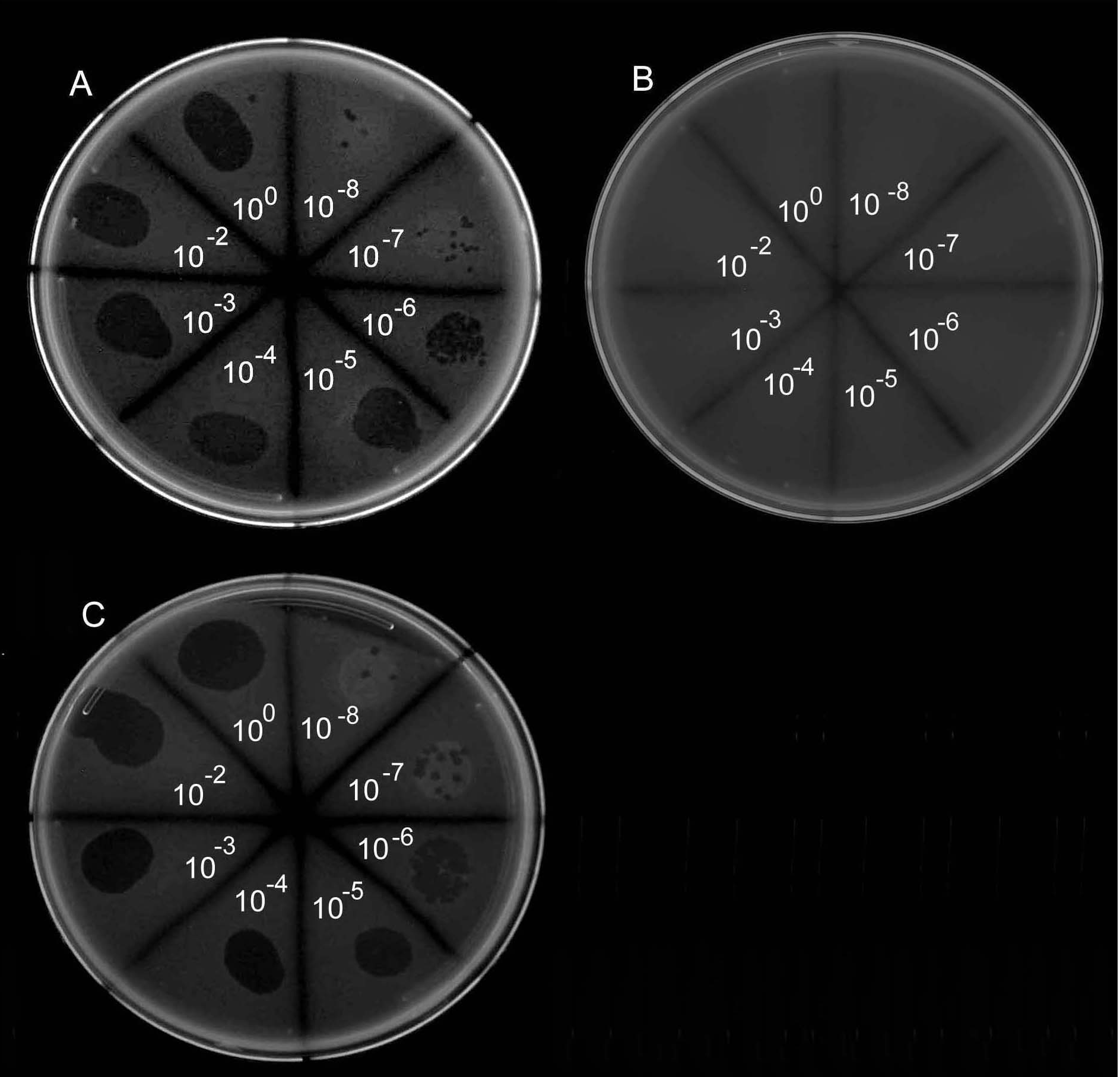

Supplement: Figure S1 — Trans -complementation of L-413C phage receptor defect. A, B, and C: L-413C plaquing on Y. pestis CO92 Pgm−, CO92 Pgm− waaL, and CO92 Pgm− waaL (pWaaL), respectively. (TIF) [file pone.0025486.s001.tif]
