# Supplementary material for: Bacteriophage-Resistant Mutants in Yersinia pestis: Identification of Phage Receptors and Attenuation for Mice
Source: PLoS One. 2011 Sep 28;6(9):e25486. doi: 10.1371/journal.pone.0025486 (PMC3182234; doi:10.1371/journal.pone.0025486)
Supplement: Table S1 — Primers for mutagenesis of Y. pestis LPS genes and verifying the sizes of amplicons. Notes: a50-bp flanking sequences of Y. pestis chromosomal DNA providing site-specific insertion of kanamycin cassette are shown in lower case type. bF1 and R1 primers targeted Y. pestis DNA on the gene flanks and provided amplification of a fragment with changed size due to the replacement with the Kmr gene. cInt (internal) primers amplified the corresponding intact Y. pestis gene and were used to exclude gene duplications and emergence of merodiploids. dThe kan-start and kan-stop primers targeted the Kmr gene and were used to amplify the DNA novel joints with corresponding F1 and R1 primers, respectively. (DOCX) [file pone.0025486.s002.docx]

**Table S1.** Primers for mutagenesis of *Y. pestis* LPS genes and verifying the sizes of amplicons

| Primer designation | Purpose | Primer sequence^a^ |
| --- | --- | --- |
| *lpxM*-F | Mutagenesis | 5'-gtgtcttctgtaatgatgattagcccaccgccaattctaagagtttccccGCACAGTCGTGATGGCAAG-3' |
| *lpxM*-R | Mutagenesis | 5’-tacattgaatggtgctgtatttaaccgccctcttgaacggttatcacaccCCCGTCAAGTCAGCGTAATG-3’ |
| *lpxM*-F1^b^ | Verification | 5'-TGAGTGGTAAAGATCGCAGTGAG-3' |
| *lpxM*-R1 | Verification | 5'-CGGTGAGGATAGGCAGAGGAG-3' |
| *lpxM*-Int-F^c^ | Verification | 5'-GCTCGCTTATATTCCACCCA-3' |
| *lpxM*-Int-R | Verification | 5'-AACAACTCCACTTCCTCATTCATC-3' |
| *yrbH*-F | Mutagenesis | 5'-cctacagcgtgaaagatgacggtaataccaacattgggcggatattaagtGCACAGTCGTGATGGCAAG-3’ |
| *yrbH*-R | Mutagenesis | 5’-atcactgactgatagcattgttcgcggcgctcattgactcaactaaactgCCCGTCAAGTCAGCGTAATG-3’ |
| *yrbH*-F1 | Verification | 5’-GTGATTATTGGGTGATGCTCG-3’ |
| *yrbH*-R1 | Verification | 5’-GGGATTCATTCGTTTGCTGC-3’ |
| *yrbH*-Int-F | Verification | 5’-GTGATGGGAATGGGCAAATC-3’ |
| *yrbH*-Int-R | Verification | 5’-GCCAATATGTTCGGAGGGACT-3’ |
| *waaA*-F | Mutagenesis | 5’-gcccagatatggtattattcttaataatccatataaatgaaattgatagaGCCACGTTGTGTCTCAAAATCTC-3’ |
| *waaA*-R | Mutagenesis | 5’-gcttcattcttggttatcatcaccaccgacagacgttttttggtacccatCGTCAAGTCAGCGTAATGCTC-3’ |
| *waaA*-F1 | Verification | 5’-ATTGCCGTGATTTCCGAGC-3’ |
| *waaA*-R1 | Verification | 5’-GTTGTAGCGATATTGGTGATGAGG-3’ |
| *waaA*-Int-F | Verification | 5’-ATCGCCGTAAGATCCCACTG-3’ |
| *waaA*-Int-R | Verification | 5’-TTCAACCAAACTACCGCCAAC-3’ |
| *hldE*-F | Mutagenesis | 5’-gggatgggtgcattcggggttaataacaactcaggttggggggcaaatgtGCCACGTTGTGTCTCAAAATCTC-3’ |
| *hldE*-R | Mutagenesis | 5’-tttaacccacccactgccacatccttatgcgccagcgctctggcgctgacCGTCAAGTCAGCGTAATGCTC-3’ |
| *hldE*-F1 | Verification | 5’-CGCTCACCCGTGCTTATGTC-3’ |
| *hldE*-R1 | Verification | 5’-GCCACCAACGCTATTACCAG-3’ |
| *hldE*-Int-F | Verification | 5’-CCGTAACCAGCAGTTAATCCG-3’ |
| *hldE*-Int-R | Verification | 5’-CCAATCCACCGCTTCCAAC-3’ |
| *waaF*-F | Mutagenesis | 5’-ggctcaatcgctcagtctaaatcacttgctgcaaggaattgataaacggtGCACAGTCGTGATGGCAAG-3’ |
| *waaF*-R | Mutagenesis | 5’-gtatgtaaaacatcgcccattgacgaggttttaacgattaatacgtgcatCCCGTCAAGTCAGCGTAATG-3’ |
| *waaF*-F1 | Verification | 5’-TTGGCTGAACCCTTCTTCTACC-3’ |
| *waaF*-R1 | Verification | 5’-TTCCAGGCTGTCGCTGATG-3’ |
| *waaF*-Int-F | Verification | 5’-GGGTTGGCGATATGATGATG-3’ |
| *waaF*-Int-R | Verification | 5’-GACTGAGCATTCTTGCGTAGC-3’ |
| *waaL*-F | Mutagenesis | 5’-tgggtcagtcgctttatttttcctaaccagctcgagctgagaaaattatgGCCACGTTGTGTCTCAAAATCTC-3’ |
| *waaL*-R | Mutagenesis | 5’-tcgagtcatcggggtttgtcagcattctgaatgtgtattgcgtctctctgCGTCAAGTCAGCGTAATGCTC-3’ |
| *waaL*-F1 | Verification | 5’-GATGTCATAGACGCAGTTGATTCC-3’ |
| *waaL*-R1 | Verification | 5’-AAATGTCAGCGACGGAGAGAAC-3’ |
| *waaL*-Int-F | Verification | 5’-GCAGCATAGCAGCGTTACTCTTAG-3’ |
| *waaL*-Int-R | Verification | 5’-TCAGACGAATCCAGCAGAAATG-3’ |
| *wabD*-F | Mutagenesis | 5’-agagcggcgtgatttttcgcggtcttatcacgcccagagattaaacaatcGCACAGTCGTGATGGCAAG-3’ |
| *wabD*-R | Mutagenesis | 5’-ttatatgtgccagggaaatacagtgtaatgaataagtatttgtaacttatCCCGTCAAGTCAGCGTAATG-3’ |
| *wabD*-F1 | Verification | 5’-CGTGGATTTCTGCTGCTGG-3’ |
| *wabD*-R1 | Verification | 5’-GGCATCGTGAATATAGTCGTG-3’ |
| *wabD*-Int-F | Verification | 5’-TCACAGTCTCGCACTACTGGAAC-3’ |
| *wabD*-Int-R | Verification | 5’-CATAGGAACCCATCAAGAATGAG-3’ |
| *wabC*-F | Mutagenesis | 5’-acatataaggtttaattaagccctgaagcaattaatgtgaatggacttaaGCACAGTCGTGATGGCAAG-3’ |
| *wabC*-R | Mutagenesis | 5’-cgacgattctgggtgataggccggtgttccggccctgatctatggcgttaCCCGTCAAGTCAGCGTAATG-3’ |
| *wabC*-F1 | Verification | 5’-CTGGCAGCAAGATGATGTCG-3’ |
| *wabC*-R1 | Verification | 5’-CCTTCTATGGCTACCGTACTAATCC-3’ |
| *wabC*-Int-F | Verification | 5’-CCAGCAGCAGAAATCCACG-3’ |
| *wabC*-Int-R | Verification | 5’-GGAAATCACCAAATCCAACTG-3’ |
| *kan*-start^d^ | Verification | 5’-GTAACATCATTGGCAACGCTACC-3’ |
| *kan*-stop | Verification | 5’-TTGATGACGAGCGTAATGGC-3’ |
