# Supplementary material for: Bacteriophage-Resistant Mutants in Yersinia pestis: Identification of Phage Receptors and Attenuation for Mice
Source: PLoS One. 2011 Sep 28;6(9):e25486. doi: 10.1371/journal.pone.0025486 (PMC3182234; doi:10.1371/journal.pone.0025486)
Supplement: Table S2 — Primers for cloning of Y. pestis LPS genes. Notes: aTwo tandem stop codons in the yrbH forward primer are underscored. Lower case letters in the rest of forward primers designate modified ribosome binding site according to recommendations of the supplier of the TOPO cloning system (Invitrogen). (DOCX) [file pone.0025486.s003.docx]

**Table S2.** Primers for cloning of *Y. pestis* LPS genes

| Primer  designation | Primer sequence^a^ |
| --- | --- |
| *yrbH*-C-F | 5’-TAATAAGCGCTGTAAAATACATTTCCC-3’ |
| *yrbH*-C-R | 5’-TCAGACAACACCGGCTCTCAG-3’ |
| *waaA*-C-F | 5’-taggaggaataataaATGCTGCTGCGTTTATACCAGGTATTACTCT-3’ |
| *waaA*-C-R | 5’-TTTTGGTACCCATTTAGTGGCTCCGT-3’ |
| *hldE*-C-F | 5’-taggaggaataataaATGAAAGTCACGCTGCCTGATTTTC-3’ |
| *hldE*-C-R | 5’-TGACTTAGCCGCGGCCGTTTTTGAT-3’ |
| *waaF*-C-F | 5’-taggaggaataataaatgaaaatactggtcatcggccctt-3’ |
| *waaF*-C-R | 5’-CGTGCATTAATCGCCCCCTTTGAC-3’ |
| *waaL*-C-F | 5’-taggaggaataataaATGACAACTTTATCACCGGCA-3’ |
| *waaL*-C-R | 5’-CTACGTTGGCAGCCGTTTACC-3’ |
